# Schoolchildren as health promoters: a community strategy for healthy eating and physical activity to reduce nutritional risk

**DOI:** 10.3389/fpubh.2026.1735714

**Published:** 2026-03-11

**Authors:** Daniela-Guadalupe González, Karla-Alejandra Bon, Adriana Caballero, Samantha Sabo, B. Camarena, Graciela Caire, Gabriela-Nallely Trejo, José-Antonio Ponce, Alma-Delia Contreras, Gloria Elena Portillo, Maria Isabel Ortega-Velez

**Affiliations:** 1 School of Medicine and Nutrition, Universidad Autónoma de Baja California, Mexicali, Mexico; 2 Faculty of Biomedical Sciences, Universidad de Occidente, Culiacán, Mexico; 3 Faculty of Nutrition and Food Sciences, Universidad de Ciencias y Artes de Chiapas, Tuxtla Gutiérrez, Mexico; 4 Center for Health Equity Research, Department of Health Sciences, Northern Arizona University, Flagstaff, AZ, United States; 5 Centro de Investigación en Alimentación y Desarrollo, A.C., Hermosillo, Mexico

**Keywords:** CBPR, health promotion, peers, promoters, school children

## Abstract

**Introduction:**

The obesity epidemic in Mexico demands sustainable solutions based on participatory strategies that actively involve the community. Community-Based Participatory Research (CBPR) is a key strategy, as it not only considers the community as a source of information but also empowers it to propose solutions tailored to local realities.

**Objective:**

This study aimed to develop and evaluate a model of schoolchildren’s health promoters (HPC) as a community communication strategy to foster better nutrition, food environment, and physical activity practices, thereby reducing nutritional risk in schoolchildren.

**Methods:**

Health promotion activities were implemented in parallel: in program schools (through child promoters or HPC) and in reference schools (using conventional methods, such as talks and brochures). Both quantitative data (anthropometrics, body composition, diet, and physical activity) and qualitative data (focus groups and interviews with parents, teachers, and principals) were collected in 36 public elementary schools across Hermosillo and Mexicali (Northwest Mexico), as well as Tuxtla Gutiérrez (Southeast Mexico), reaching a total population of approximately 10,800 children.

**Results:**

The results showed that body fat percentage was significantly lower in children after the HPC program compared to the reference group (33.9 ± 10.4 vs. 35.7 ± 9.7, *p* = 0.001); also, total cholesterol (18.93 ± 41.51 vs. 1.01 ± 41.45; *p* = 0.019), HDL cholesterol (14.63 ± 11.54 vs. 6.56 ± 15.56; *p* = 0.005), and the Healthy Eating Index (HEI) (3.5 ± 18.1 vs. − 3.4 ± 15.7; *p* = 0.026), improved with the participatory model. The main environmental factors identified as barriers were the unhealthy food supply at school and home, the lack of promotion for regular physical activity, and family economic limitations.

**Discussion:**

Despite the challenges children face in exercising their agency in traditional interactions, this study suggests that a child-promoter model is more effective than conventional nutrition education and that involving the school community in activities to improve their health yields promising results in reducing nutritional risk.

## 1 Introduction

Obesity in Mexico is currently one of the main public health problems. Addressing requires a systemic, complexity-based approach (1, 2). Public policies, such as a tax on sugary drinks and a front-of-pack warning labeling system, have been implemented as part of a public health policy. However, nutrition education remains underrepresented in the national strategy, and the observed impacts are still modest (3–5). On the other hand, individual-centered strategies need to be complemented by approaches that address the contexts in which people live, such as their homes, communities, workplaces, and schools, to enhance national public health efforts (6–8).

The school environment is recognized as a strategic space for implementing health promotion programs (9, 10). However, some studies have documented that school settings often favor the consumption of energy-dense foods and sugary beverages, as well as limited access to physical activity spaces (5, 11, 12).

Although traditional health promotion actions, such as talks, workshops, and educational programs, have proven efficient in improving healthy eating and increasing physical activity, their impact is often short-lived and relies on external human resources for their implementation (12–14). Despite these limitations, evidence also suggests that certain conditions can foster sustainable and significant changes in school health, such as active parental involvement, social support, nutrition education, and interventions that address multiple levels of children’s interaction with their environment (15–17).

On the other hand, regarding public health policies and interventions developed and implemented worldwide to address childhood obesity, evidence suggests that those based solely on traditional education methods and behavioral individual changes may not be sustainable in the long term. It is increasingly clear that family and community participation in obesity prevention strategies is crucial for their long-term success (18–20).

In this matter, the WHO proposes that addressing childhood obesity through prevention is necessary, with community participation within the framework of Community-Based Participatory Research (CBPR), and by identifying community leaders as a key factor in achieving agreements among different actors in the health promotion process (21). This way, networks of health promoters within the CBPR framework can develop strategies and actions that promote more sustainable and healthy lifestyles. Recently, Hernandez (22), in a study involving schoolchildren in Central Mexico, discusses that “food and nutrition education generate food practices guided by technical knowledge, but also by cultural guidelines based on the feelings involved in self-care and the care of others, in trust in one’s own actions and those of others, in respect, in empathy, and in collaboration for a supportive and committed coexistence in building healthy environments.” Peer encouragement of healthy diet and physical activity practices involves not only the transmission of knowledge but also messages from trusted peers within a specific cultural and social environment, emphasizing the importance of context in health promotion.

Furthermore, compared to other research frameworks, participatory approaches to health promotion are cost-effective since they require a small number of external agents, unlike traditional nutrition education programs, which often necessitate the involvement of numerous external agents in the community over an extended period (23). Within the above context, this study hypothesized that, through a community-participatory approach, elementary school children will be a more effective communication channel with their peers than traditional nutrition education programs delivered by an external team. Therefore, this study aimed to develop and evaluate a model involving schoolchildren as health promoters (HPC), using peer communication as a channel to promote nutrition, a healthy food environment, and physical activity practices that help reduce children’s nutritional risks.

## 2 Sample and methods

### 2.1 Study design and sample size

The project involved two types of samples: children trained as health promoters (*n* = 298) and children who received the program (*n* = 50 per school) in three Mexican cities. The design included schools selected through convenience sampling and children randomly assigned to evaluate program outcomes. Six schools per region (18 total) were selected from urban areas with medium and high marginalization indices in Hermosillo, Sonora, and Mexicali, Baja California, in Northwest Mexico, and in Tuxtla Gutiérrez, Chiapas, in Southwest Mexico. The selection criteria for the republic’s states were based on convenience. We aimed to include Northern and Southern Mexican states, given the cultural differences in community practices and food environments, to assess whether the HPC Model would be effective in diverse settings. Mexicali and Hermosillo are both northern states, but Mexicali is a border city, which provides insight into other cultural traits that primarily affect food practices. The number of schools in the reference group was the same (*n* = 18). The study protocol was submitted and approved by the Ethics Committee at Centro de Investigación en Alimentación y Desarrollo, A. C., document number CEI/021/2021.

### 2.2 Selection of schoolchildren as health promoters

Children with potential to act as health promoters were selected using the CBPR methodology (23, 24). Five puppet theater sessions were held in each school for groups in grades 3 through 5. The sessions explored the characteristics of health promoters, including friendliness, empathy, solidarity, and responsibility (25), as well as oral communication skills (25, 26). Subsequently, the students nominated 20 children per school who, in their view, met the requirements discussed to become health promoters. The selected children were asked to obtain their parents’ informed consent, which was then signed.

### 2.3 Training children as health promoters (HPC)

#### 2.3.1 Pedagogical theoretical foundations of educational intervention

The pedagogical foundation of this study is rooted in symbolic interactionism as an epistemological approach and in sociocultural theory. Educational intervention acknowledges that meaning is created through interaction and is grounded in how individuals act in accordance with the meanings they attribute to things, which emerge from social interaction and are continually modified. The school is a key space for constructing identity and reality through language and symbols. Thus, the guiding principle of the intervention begins by identifying students’ perceptions through their interactions within their context, considering Lev Vygotsky’s contributions to the field of education. These contributions include highlighting social interaction as the driving force of learning, the Zone of Proximal Development (ZPD) as a guide for teaching, the importance of language as a tool for thought, and the crucial role of cultural context in the construction of knowledge. The intervention also promotes collaborative learning and the role of the “more expert peer” to enhance skills (27).

Once the health promoters were selected, content was developed based on a previous study’s needs assessment of nutrition education (12). Ten interactive workshops with HPC, each lasting approximately 45 min, were conducted after school hours. These workshops covered topics such as the Healthy Eating Plate, appropriate portion sizes, healthy lunch options, front-of-packaging warning labels, physical activity, and adequate use of electronic devices. During the training, the children proposed strategies for delivering each health promotion theme related to their topics, and the research team recorded them.

### 2.4 Design and implementation of community strategies

After the training, HPC served as communication channels to their peers, proposing and developing strategies and actions to promote healthy eating and physical activity, and modifying environments that were conducive to these behaviors. Implementation through the HPC with peers took place from March 2023 to April 2024. HPC talked to their peers about healthy eating and physical activity in the classroom (from 1st to 6th grade), during the school’s ceremonial events (such as the flag-raising ceremony every Monday), on posts on the school’s bulletin board, during midday recess, and through small talks in the school hallways, as well as during sports tournaments, from one to three times a week.

The research team conducted a process evaluation through focus groups with children, parents, and key stakeholders (teachers, school principals, and school store owners), which began midway through the process and continued until the end. In addition, the research team held weekly meetings with HPC to address any questions or concerns and to determine what materials the children would need for their next activity. Along with implementing the HPC strategies, the research team had monthly traditional nutrition education talks in the reference schools. The research team distributed banners related to the talk’s content and displayed them throughout the school year in the 18 schools that formed the reference group.

### 2.5 Nutritional risk assessment

For nutritional risk assessment, weight, height, waist circumference, and body fat (measured by Bioelectric Impedance Analysis) were assessed in children in the HPC program and the reference group at the beginning and end of the program. In addition, a multiple pass 24-h recall (28, 29), physical activity and socioeconomic questionnaires (30, 31), and biochemical indicators (total cholesterol, fasting glucose, triglycerides, HDL-cholesterol, systolic and diastolic blood pressure) (32–36) were assessed in a subsample of both children in the HPC program and the reference group. Sedentarism was evaluated by the time children spent in front of a screen (TV, tablet, or phone) and classified as less than or more than 2 h (37). Measurements were taken at the beginning and end of program implementation.

### 2.6 Qualitative data

Using Realistic Theory as a guide, we explored the mechanisms, contexts, and outcomes of the HPC program through interviews with key actors (principals, teachers, and food vendors), interviews with HPC, focus groups with HPC parents, and direct observations of the school environment. The last one was based on the 2014 guidelines for the sale of food in school establishments. We added a question about physical activity installations (38) The guide included a brief assessment tool (six items) of the school environment related to food (breakfast area and healthy food vending, access to pure water, and the absence of street vendors or lunch purchasing in nearby food stores), as well as physical activity installations (shaded sports court) in each HPC program school. The sample for the focus groups was determined using the theoretical saturation method (39). Each interview guide underwent validation through triangulation, with two principal investigators and two collaborating researchers participating.

### 2.7 Data analysis

Quantitative data analysis was performed using descriptive statistics, including means, standard deviations, and Chi-square analysis for proportions. To evaluate the effect of the intervention on BMI, waist circumference, and percent body fat, linear mixed-effects models were used. These models included the post-intervention value of each outcome as the dependent variable, and the intervention group as a fixed effect. School was included as a random effect to account for the study’s clustered design. For reference, analyses using ANCOVA, adjusted for age and sex and accounting for clustering, were also conducted. Additionally, sensitivity analyses including random intercepts at the city level were performed to explore potential contextual effects across study locations.

Dietary data were analyzed using the Healthy Eating Index (40–42), based on the dietary guidelines for the Mexican population (43). A comparison of non-normally distributed data was performed using the Mann–Whitney *U* test (44). Effect sizes were calculated for biochemical data as Cohen’s *d* (mean difference divided by the pooled standard deviation).

Regarding the qualitative data analysis, the interviews and focus groups were transcribed verbatim and analyzed based on thematic categories using the Realistic Evaluation Theory, identifying the Context, Mechanism, and Outcomes (CMO) configuration that triggered positive changes (45, 46). The NVivo 10 program was used to organize the data.

## 3 Results

Table 1 presents the sample sizes for measurements and indicators among children who received the HPC program and the reference children, as well as the total number of children trained as health promoters at the beginning and end of the program. The dropout rate among children who received the program was due to everyday family adjustments, such as moving to a new neighborhood. Additionally, older children (those in 6th grade) continued their education in Middle School, which in Mexico means attending a different school. There were also issues with irregular school attendance due to family problems, particularly in Tuxtla Gutiérrez, in Southern Mexico. Another factor contributing to participant attrition was the requirement for blood samples. Although parents and children initially agreed to provide a second sample, during the project’s follow-up stage, many ultimately declined to do so. The dropout rate among HPC was primarily due to the workload and exams at the end of the school year, which limited the participation of some who were concerned about their academic performance. Most children in both the HPC and the reference groups belonged to households with medium or low socioeconomic status (90% in the HPC and 91% in the reference group).

**Table 1:** Sample sizes.

Measurement	HPC program		Reference	
	Before program	After program	Before program	After program
Anthropometry and body composition	622	414	531	386
Diet	146	71	102	47
Physical activity	149	67	91	41
Socioeconomic	146	70	100	41
Biochemical indicators	155	80	118	44
Total children as health promoters	298	142		

### 3.1 Quantitative analysis

Taking into account that our study’s main objective was to develop and evaluate the effectiveness of a participatory, community-based model (Health Promoters Children, HPC) in reducing nutritional risk, particularly childhood obesity, Table 2 presents the results of anthropometric measures and comparison of indicators among schoolchildren participating in the HPC program and those from the reference group prior to the program’s implementation. There were no significant differences in most variables, except for the mean age.

**Table 2:** Anthropometric, body composition, and percentage of girls among schoolchildren who received the HPC program and in the reference schools, before program implementation.

	HPC *n* = 622	Reference *n* = 531	*p*-value
% Girls (n)	52 (324)	50 (264)	
Variable	Mean ± SD	Mean ± SD	
Age	9.03 ± 1.5	8.98 ± 1.6	0.005
Weight (kg)	33.1 ± 11.8	34.0 ± 12.3	0.058
Height (cm)	132.2 ± 11.4	132.4 ± 11.4	0.184
Z BMI/age	0.62 ± 1.85	0.89 ± 1.9	0.782
Waist circumference (cm)	64 ± 11.3	65.4 ± 11.8	0.075*
% Body Fat	30.2 ± 10.3	30.8 ± 9.7	0.197**

* Adjusted by age, sex, height. ** Adjusted by age and sex.

Table 3 presents the data after program implementation. When comparing nutritional risk indicators between HPC program children and children in reference schools, using an ANCOVA adjusted for age and sex, without accounting for the clustered design, no significant differences were found in BMI or waist circumference. However, the body fat percentage was lower in children in the HPC school program (*p* = 0.001). Nevertheless, using linear mixed methods and including schools as a cluster, the differences in percentage body fat were no longer significant (*p* = 0.197). Furthermore, in sensitivity analyses specifying random intercepts at the city level, the difference in body fat percentage remained statistically significant (*p* = 0.023).

**Table 3:** Post-intervention comparison of BMI, waist circumference, and percent body fat between schoolchildren in the HPC program and reference schools.

	Schools with HPC *n* = 415	Reference schools *n* = 386	*p-*value		
% Girls (n)	51 (212)	47 (180)			
Variable	Mean ± SD	Mean ± SD			
Weight (kg)	39.7 ± 12.4	40.2 ± 14.2			
Height (cm)	140.8 ± 10.6	140.2 ± 11.1			
Z BMI/age	1.0 ± 1.87	1.1 ± 2.0	0.086*	0.770**	0.385***
Waist circumference (cm)	69.6 ± 12.0 (*n* = 412)	68.3 ± 12.6	0.063*	0.316**	0.174***
% Percent body fat	33.9 ± 10.4 (*n* = 410)	35.7 ± 9.7 (*n* = 384)	0.001*	0.197**	0.023***

*Adjusted by age, sex, and height for waist circumference. **Adjusted by age, sex, and including school as cluster. ***Adjusted by age, sex and including city as cluster.

As one of the primary mediators of nutritional risk and a key aspect of intervention design, dietary data were analyzed using the Healthy Eating Index (HEI). The results showed that 81% of schoolchildren in both groups still require some healthy dietary changes before and after the HPC program. However, the comparison of changes in HEI after the HPC program showed better results in children receiving the HPC program (3.5 ± 18.1) compared to the reference group (−3.4 ± 15.7), *p* = 0.026; significant positive changes were observed in fruit intake (*p* = 0.020) and the overall diet variety (*p* = 0.033), Table 4.

**Table 4:** Comparison of the HEI and HEI components among schoolchildren who received the HPC program and those in the reference schools.

Indicator	Δ Schools with HPC (*n* = 60)	Δ Reference Schools (*n* = 36)	*p-value
	Mean ± DS	Mean ± DS	
Healthy eating index			
Cereals	−0.2 ± 2.3	0.4 ± 3.6	0.396
Vegetables	0.3 ± 4.1	−0.5 ± 2.6	0.160
Fruit	1.2 ± 5.4	−1.2 ± 5.5	**0.020**
Dairy	−2.4 ± 3.8	−1.7 ± 4.1	0.430
Meats	0.2 ± 2.0	−0.8 ± 4.2	0.174
Total lipids	0.7 ± 4.9	−1.0 ± 4.7	0.111
Saturated fats	1.1 ± 5.0	0.3 ± 4.8	0.431
Sodium	0.6 ± 5.3	0.3 ± 4.9	0.976
Sugars	2.1 ± 3.9	1.9 ± 3.5	0.776
Variety	−0.1 ± 2.3	−1.1 ± 2.8	**0.033**
Total score	**3.5 ± 18.1**	**−3.4 ± 15.7**	**0.026**

X ± SD: Mean ± Standard Deviation. *Non-parametric Mann–Whitney *U* test.

On the other hand, sedentarism analysis showed that there were no significant differences in the proportion of active children (less than 2 h in front of a screen) (61 vs. 79 for children in the HPC program and 57 vs. 77 for children in the reference group), despite 18 and 20 percentage points of difference (*p* = 0.31).

Regarding the food and built environment in the HPC schools’ program, data showed that schools in Mexicali City achieved 80% of the desirable score for school environment, followed by Hermosillo (47%) and Tuxtla Gutiérrez (39%).

As additional explanatory effects on health risks related to diet and physical activity, Table 5 compares changes in three of the biochemical indicators considered risk factors for the presence of metabolic syndrome, that showed small to medium effect sizes: fasting glucose (−16.88 ± 14.12 vs. -9.63 ± 19.53, *p* = 0.041; d = −0.44), total cholesterol (−18.93 ± 41.51 vs. 1.01 ± 41.45, *p* = 0.019; d = −0.48), and high-density lipoprotein cholesterol (14.63 ± 11.54 vs. 6.56 ± 15.56, *p* = 0.005; d = 0.61). For fasting glucose, the decrease in children having the HPC program was 16 units, while the reference children decreased by 9.63 units; for total cholesterol, the decrease was 18.9 units vs. 1 unit, and in the case of high-density lipoprotein cholesterol (HDL), an increase is desirable, as it is considered protective (47). Thus, the number of children participating in the HPC program increased by 14.6 units, while the number of reference children increased by 6.5 units.

**Table 5:** Comparison of changes in biochemical markers in children in the HPC program and reference program before and after program implementation.

Indicator	Δ Schools with HPC (*n* = 65)	Δ Reference schools (*n* = 39)	*P-*value*	Effect** size
	Mean ± DS	Mean ± DS		
Cholesterol (mg/dL)	−18.93 ± 41.51	1.01 ± 41.45	0.019	−0.4
Triglycerides (mg/dL)	−20.81 ± 44.84	−18.66 ± 61.0	0.865	−0.04
HDL – C (mg/dL)	14.63 ± 11.54	6.56 ± 15.56	0.005	0.61
Glucose (mg/dL)	−16.88 ± 14.12	−9.63 ± 19.53	0.041	−0.44
Diastolic blood pressure	10.92 ± 14.97	6.24 ± 12.14	0.412	0.33
Systolic blood pressure	6.33 ± 10.9	2.10 ± 13.15	0.788	0.3

*Adjusted by age and sex. ** Cohen’s *d*.

### 3.2 Qualitative analysis

On the other hand, through qualitative analysis of focus groups and interviews with key stakeholders (teachers, parents, principals, and school store managers), contexts and mechanisms that contribute to children’s intentions to change and changes in their eating and physical activity practices were identified. The key contexts were aspects of the school environment (unhealthy food environment, lack of initiatives for regular physical activity, seven quotes),

*Yes, they are still selling junk food. When we pass by the school store, we see them [our peers] coming out with chips, candy, and instant soups. There is fruit, but they do not buy it.* — Schoolchild, school in Mexicali.

*My physics teacher rarely attends; he only comes when we are going to play soccer, and then I do not like the class he gives us because it is just about playing soccer. If we are here and he is over there, even on the other field, with his phone charging, he ignores us:* Schoolchild, 6th grade, school in Hermosillo.

Moreover, the students’ interaction with their families (as evidenced by family support to improve lunches and meals at home, as indicated in 23 quotes).

*My child must now bring a healthy lunch. Before, it was some cookies or some Sabritas or [something] bought here [school], and now he brings his sandwich, his quesadilla, his bean taco…* — Mother of a schoolchild at Hermosillo.

*My daughter used to eat many chips [Sabritas] before, but now she comes and tells me she does not anymore because it hurts her stomach, or she tells me, 'Mom, do not eat that, it is going to make you sick.* — Mother of a schoolchild in Tuxtla Gutiérrez.

the student’s interaction with teachers and the school (reinforcement of nutrition education activities, the HPC program as a supporter, six quotes),

*I think it [the HPC Program] helps us empower students in reality; it helps us expand their knowledge, and it also allows us, in a certain way, to provide feedback on what we do in class.* — School Principal, a school in Hermosillo.

*The parents will be coming to school for a periodic reunion. I will lead an activity where the children, along with the health promoters, visit each classroom. Perhaps that would be a way, because right now, if a teacher tells their group that the children should eat breakfast here and that we should instill healthy eating habits at home, but if they hear it from the children themselves, maybe it will be different.* —Principal of a school in Mexicali.

Furthermore, the family economic environment (mainly in the city of Tuxtla Gutiérrez, in southern Mexico, as noted in three quotes).

*It could be the parents' economic situation, or perhaps a lack of organization, because sometimes the mom, instead of giving them breakfast, sends them a little juice box or gives them money to buy something. What does the child do? They buy Sabritas [chips], they buy other things. So, it is like something is lacking [income].* —Teacher, School in Chiapas.

*The main problem is the economic level, as the children eat what their parents barely have at home. No, they do not have the means to have a balanced diet.* — Teacher, School in Chiapas.

The primary mechanisms of change were children’s demands for changes in eating and physical activity at home, which reinforce their importance as agents of change (14 quotes).

*If the children are doing it, it is because their parents are collaborating with them. The children are promoters, and at home, they are also following their father's example. The parents are really helping them; they are getting involved because we continually see that the children are bringing healthy lunches and fruit…* — Teacher, school in Hermosillo.

*I am surprised because there are promoters who take on the role of promoting healthy eating, suggesting what to eat, what to nourish themselves with during recess, and what not to eat.* — Teacher, school in Mexicali.

In addition, the trust-based relationship between the facilitating group (researchers and nutrition students) and the children, as well as their parents, facilitated behavior change and training (as indicated in four quotes).

*For example, when you come and share with the students, I know that this is about child promoters, but that you, as professionals, also share the topic [during training], and that you come, they have much respect for you.* — School Principal, a school in Hermosillo.

*They have done their part [the researchers and nutrition students]. The children have been focusing on it well; they are stimulated and motivated, and you can see it. As soon as the group arrives, they run right over, and the children are right there with you.* — School Principal, a school in Mexicali.

Among the main barriers to healthier change were the school food environment (both inside and outside the school), the difficulties some children face in being heard, and the need for greater parental and teacher involvement (as indicated in 20 quotes).

## 4 Discussion

This research aimed to develop and evaluate a model involving schoolchildren as health promoters, using peer communication as a channel to promote nutrition, changes in the food environment, and physical activity practices that help reduce children’s nutritional risks. In that context, body composition, measured as body fat percentage, appears to be an indicator of change associated with the HPC program at the individual level, suggesting changes in schoolchildren’s dietary practices at home, which is supported by the qualitative analysis of focus group discussions. However, when we analyzed our data with the school as a cluster of children’s interactions with a given food and built environment, the results for body fat were no longer significant. On the other hand, when changes that could be attributed to each city as a cluster were introduced, differences persisted (48, 49). Our qualitative data analysis indicates that differences in the school environment, as well as teachers’ and school principals’ support for HPC activities, may be affecting the program’s effectiveness. On the other hand, the HPC program seems to work well for all cities included in this study.

On the other hand, overall improvements in HEI, diet variety, and fruit consumption among children who received the HPC program show promise; however, the high percentage of children who still require changes in their daily nutrition indicates the challenges faced by low- to medium-income families in following a healthy diet. The food environment is also a contributing factor to unhealthy diets, as demonstrated by other national and regional studies (5, 22, 50, 51).

Qualitative data expanded our understanding of changes in the study-specific context. The data reveal how families face economic barriers and challenges in the food environment when trying to provide their children with a healthier diet, particularly in southern Mexico. However, within their means, dietary changes and physical activity facilitate an intention to change, as promoted by their children. The study by Hernández J.C. in Central Mexico demonstrated that intervention strategies targeting teachers and cooks were more effective in improving the school food environment. Recognizing these practical strategies can inspire public health researchers to prioritize such interventions, reinforcing their vital role in shaping healthier school environments (22). Our own research also underscores the importance of regulatory compliance, as it is crucial to the sustainability and effectiveness of healthy food initiatives, making educators and stakeholders feel that their efforts are essential (12).

An additional exploration of an effect on health risks related to obesity and nutrition strengthened by the results of biochemical indicators such as fasting glucose, total cholesterol, and triglycerides, which were lower for children who participated in the HPC program compared to children in reference schools; as well as high-density cholesterol (HDL), which improved better for children in the HPC program than those in the reference group. These results, together with those on body composition, contribute to other research that suggests body composition indicators are more sensitive to educational interventions for obesity prevention (52). Other authors have noted that changes in biochemical indicators can be more readily observed when improving diet and physical activity in children (53, 54).

Overall, it seems that having children actively participating through an HCP program has been more efficient than traditional nutrition education programs to diminish nutritional risk; some studies and systematic reviews in recent years have shown that different programs that include varied quasi-experimental, randomized clinical, multi-component designs, show small or non-existent results in the nutritional risk of children in different countries (16, 17, 55). The authors discussed the need to address obesity prevention as a complex health problem that extends beyond individual and family behavioral change; in this regard, the food, social, and built environments serve as mediators of individual, family, and community agency and change (8).

On the other hand, this study yielded promising results, despite the ongoing impact of the pandemic, which has affected social, environmental, family, and school conditions in the daily lives of populations worldwide, as well as in the context of this study (8, 56).

Within the principles of community-based participatory research (CBPR), developing research that incorporates community participation, not only as providers of information and recipients of health promotion actions, but also as members who propose solutions in accordance with their local realities, has been supported by other researchers in the public health area (57). In this context, the identification and training of health promoters among schoolchildren were intended to foster community (schoolchildren and families) ownership of health promotion and the sustainability of the changes achieved. The development of self-efficacy and the practice of health promotion communication with peers, guided by the principles of CBPR, and highlighting social interaction as the driving force of learning, influenced changes in children’s eating habits and an intention of change in sedentary behavior through proposals that create a healthier food environment and promote physical activity in their homes and schools.

CBPR proposes transforming knowledge generation by transferring some control to the community, which is aware of the problems in its local context and capable of proposing strategies to address them. In addition, it has been shown that it can serve as a platform for sustainable strategy development, especially in marginalized communities, by encouraging community empowerment to propose sustainable solutions to problems within their local context (58). In this way, the focus of CBPR is on community inclusion, not only as recipients of health promotion programs but also as active members proposing solutions to problems within their local context. This way, strategies are considered in which the community actively seeks solutions that can become public policies (57, 59–61).

## 5 Strengths and limitations of this study

One of the main strengths of this study is recognizing schoolchildren’s agency in selecting their peers to become health promoters and in maintaining confidence in their own peer communication strategies throughout the program implementation, despite the research team’s traditional nutrition education practices. Regarding data collection, before the program was implemented, the children’s ages were distributed across all school grades; however, at the end of the program, the age range of the children we measured was skewed towards older elementary school students. This could confound our results on waist circumference, as pubertal changes may accelerate growth in some children aged 9–12 (62). Also, qualitative data indicated intentions and actions to increase physical activity that our quantitative study did not identify. We also acknowledge that this study did not include a quantitative calculation of the durability of the intervention’s effect; however, a follow-up study is underway.

## 6 Conclusion

This study supports other research regarding the need for more participatory and inclusive nutritional health promotion. It demonstrates that even in an adverse food environment and among economically challenged populations, the CBPR approach to nutrition education has the potential to achieve meaningful reductions in nutritional risk, though these remain small and promising. This potential should inspire future research to emphasize changes in household and community food environments alongside nutritional health promotion.

## Data Availability

The raw data supporting the conclusions of this article will be made available by the authors, without undue reservation.
